# The crowns have eyes: multiple opsins found in the eyes of the crown-of-thorns starfish *Acanthaster planci*

**DOI:** 10.1186/s12862-018-1276-0

**Published:** 2018-11-12

**Authors:** Elijah K. Lowe, Anders L. Garm, Esther Ullrich-Lüter, Claudia Cuomo, Maria I. Arnone

**Affiliations:** 1 Biology and Evolution of Marine Organisms, Stazione Zoologica Anton Dohrn, Villa comunale, 80122 Naples, Italy; 2 Marine Biological Section, University of Copenhagen, Copenhagen, Denmark; 3 Museum für Naturkunde, Berlin, Germany

**Keywords:** Transcriptomics, Asteroidea, Vision, Photoreceptors, Evolution, Echinoderm

## Abstract

**Background:**

Opsins are G protein-coupled receptors used for both visual and non-visual photoreception, and these proteins evolutionarily date back to the base of the bilaterians. In the current sequencing age, phylogenomic analysis has proven to be a powerful tool, facilitating the increase in knowledge about diversity within the opsin subclasses and, so far, at least nine types of opsins have been identified. Within echinoderms, opsins have been studied in Echinoidea and Ophiuroidea, which do not possess proper image forming eyes, but rather widely dispersed dermal photoreceptors. However, most species of Asteroidea, the starfish, possess true eyes and studying them will shed light on the diversity of opsin usage within echinoderms and help resolve the evolutionary history of opsins.

**Results:**

Using high-throughput RNA sequencing, we have sequenced and analyzed the transcriptomes of different *Acanthaster planci* tissue samples: eyes, radial nerve, tube feet and a mixture of tissues from other organs. At least ten opsins were identified, and eight of them were found significantly differentially expressed in both eyes and radial nerve, with R-opsin being the most highly expressed in the eye.

**Conclusion:**

This study provides new important insight into the involvement of opsins in visual and nonvisual photoreception. Of relevance, we found the first indication of an r-opsin photopigment expressed in a well-developed visual eye in a deuterostome animal. Additionally, we provided tissue specific *A. planci* transcriptomes that will aid in future Evo Devo studies.

**Electronic supplementary material:**

The online version of this article (10.1186/s12862-018-1276-0) contains supplementary material, which is available to authorized users.

## Background

Light carries an immense amount of information about the surroundings. Direct light from the sun, the moon, or the stars is used by various animals to set diurnal or annual clocks and to set direction during navigational tasks. Light reflected from the surroundings guides innumerous different behaviors as it provides information about objects with unprecedented details and speed. Light reception is thus widespread in the animal kingdom but interestingly the molecular machinery behind light reception shares many common features across all phyla. In most cases examined so far the first step in the phototransduction, the absorption of the photons in metazoan, is mediated by a specific protein family called opsins [1]. Opsins are seven transmembrane G protein-coupled receptors binding a chromophore, retinal, which undergoes a conformational change upon the absorption of light, thus triggering the rest of the transduction cascade. The coupling of the opsin and the G protein alpha subunit determines which phototransduction cascade, or particular function will take place [2–5]. During the last couple of decades, several molecular studies have examined the diversity of the opsin family, identifying at least three major clades (rhabdomeric, ciliary, and RGR/Go or group 4) [6]. As the number of sequenced species grow, so does the number of opsin groups, enabling to better classify of previously considered lineage specific opsins (e.g. cnidopsins). Recently, a classification has been proposed which has nine clades of opsins in total [7, 8]. While Rhabdomeric (r-opsin) and, in some rare cases, go-opsins are the primary visual opsins in protostomes (such as insects, annelids and mollusks) and ciliary opsins (c-opsins) have been identified as the primary visual opsin in vertebrates, little is known about the opsin types used for vision in early branching deuterostomes (such as protochordates and ambulacrarians). What the work has also shown is that many animals have a surprisingly high number of opsin gene copies and that they can be expressed in almost any body region or organ [9]. In many of these cases, the functions remain unknown and may well be outside light reception [10, 11].

Light reception is known from all major groups of echinoderms and is facilitated by different types of photoreceptors ranging from non-pigmented dermal photoreceptors to proper image forming eyes. One of the best studied examples of dermal photoreception is found in the brittlestar genus Ophiocoma [12, 13]. Another dermal photoreceptor system is found in sea urchins which has been suggested to support image forming vision [14, 15]. The genome has been sequenced for the sea urchin *Strongylocentrotus purpuratus* and eight opsin genes were found belonging to the opsin clades c-opsins, r-opsins, Go-opsins, peropsin, neuropsin and echinopsins A and B [7, 16]. The latter two groups were recently renamed as bathyopsins and chaopsins, respectively [8]. The r-opsin Sp-opsin4 is expressed in cells at the base of the transparent tube feet and is putatively mediating the directional negative phototaxis described for a couple of species [15, 17, 18]. The brittle star *Amphiura filiformis* has even higher opsin diversity with at least 13 gene copies [19], but here little is known about expression patterns and behavioural roles.

**Fig. 1:**
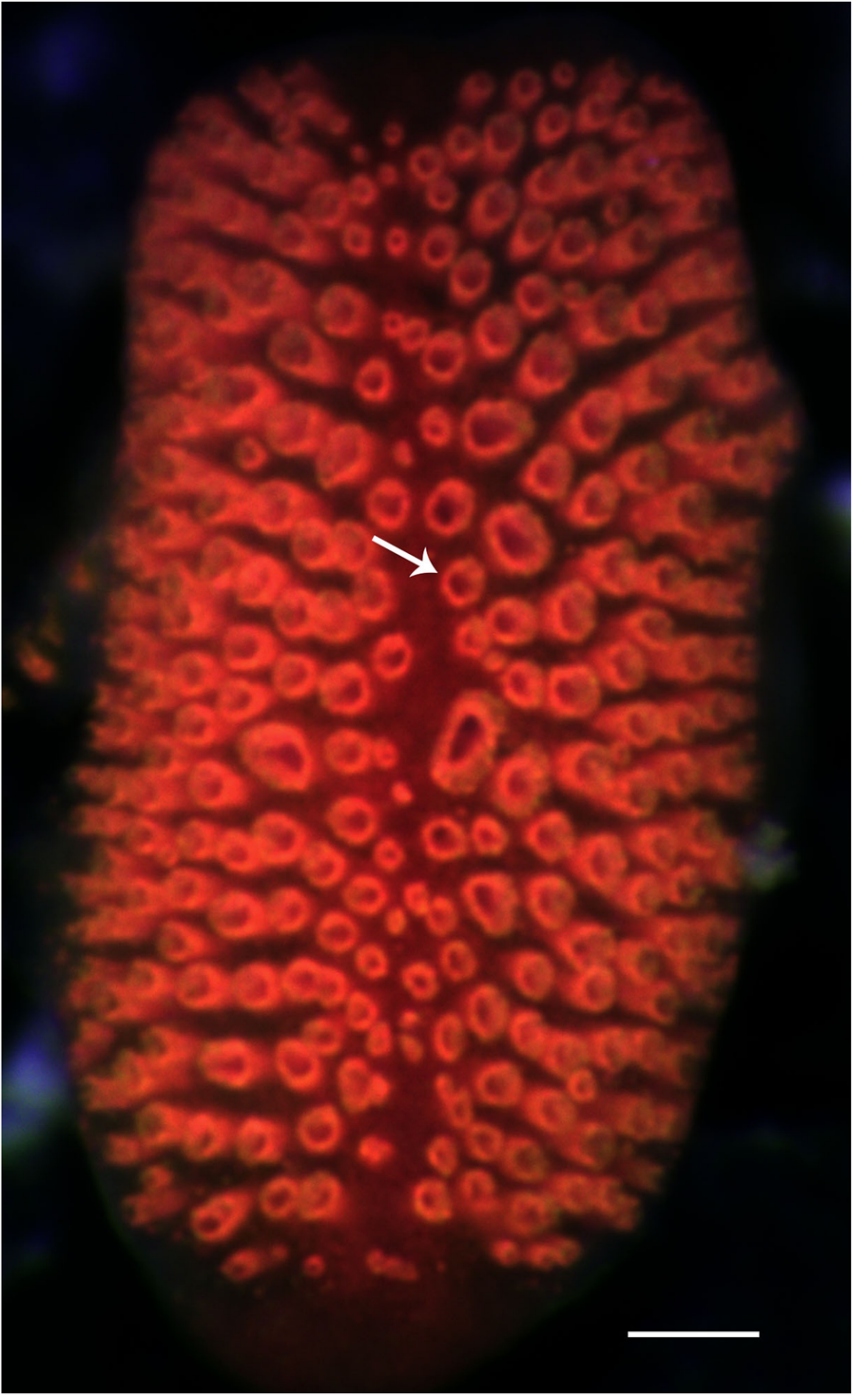
The compound eye of *A. planci*. The eyes are found on the distalmost tube foot on each arm tip and the fully grown eye has about 300 ommatidia here seen as red rings formed by the screening pigment (arrow). The eyes are image forming and each ommatidium is thought to constitute a separate pixel in the image. Scale bar = 100 μm

Both dermal photoreception and proper eyes are known from several species of starfish (Asteroidea) [20–22] but it is unknown if it is opsin based and if so whether it is the same opsin in the two systems. The eyes are found in most non-burrowing starfish species at the tip of each arm sitting at the base of the unpaired terminal tube foot as a direct extension of the radial nerve. They are compound eyes and structurally they resemble the eyes of arch clams and fan worms [23, 24] with lens-less ommatidia typically 20–40 μm in diameter (Fig. 1). Depending on species, adult specimens have 50–300 ommatidia in each eye and recent studies have shown that this supports spatial resolution in the range of 8–17 degrees used for navigation [25–27]. These studies have also indicated that the ommatidia have a single population of photoreceptors which utilize an opsin with peak sensitivity in the deep blue part of the spectrum around 470 nm.

Recently, two draft genomes of the crown-of-thorns starfish (COTS) *Acanthaster planci,*^1^ relative to animals collected from Okinawa, Japan and Great Barrier Reef (GBR), Australia, were released. These two genomes shared 98.8% nucleotide identity and were determined to be the same species [30], which is in agreement with previous classification of the Pacific ocean COTS being one species [28]. Although *A. planci* is not the first Asteroidea with an assembled genome, it is the first species with well-defined eyes to have an assembled genome. The GBR genome was released along with annotations for ~ 24,500 protein coding genes [30], and this presents an opportunity to study the mechanisms behind image forming vision in an echinoderm. There are various theories on the origin of opsins and which opsins were present in the ancestral eye [31]. Investigating a wide range of species with various types of opsin based photoreception will likely aid in the understanding of opsin origins. Here we have used tissue specific transcriptomics to investigate the differential expression of opsin genes in *A. planci*. We found ten different opsins belonging to seven different clades and by comparing expression levels in the eyes, in locomotory tube feet, in the radial nerve, and in a mixture of gonadal, stomach, and epidermal tissue, we are able to infer which opsins are most likely used in vision, in non-visual photoreception, and outside photoreception.

## Results

**Fig. 2:**
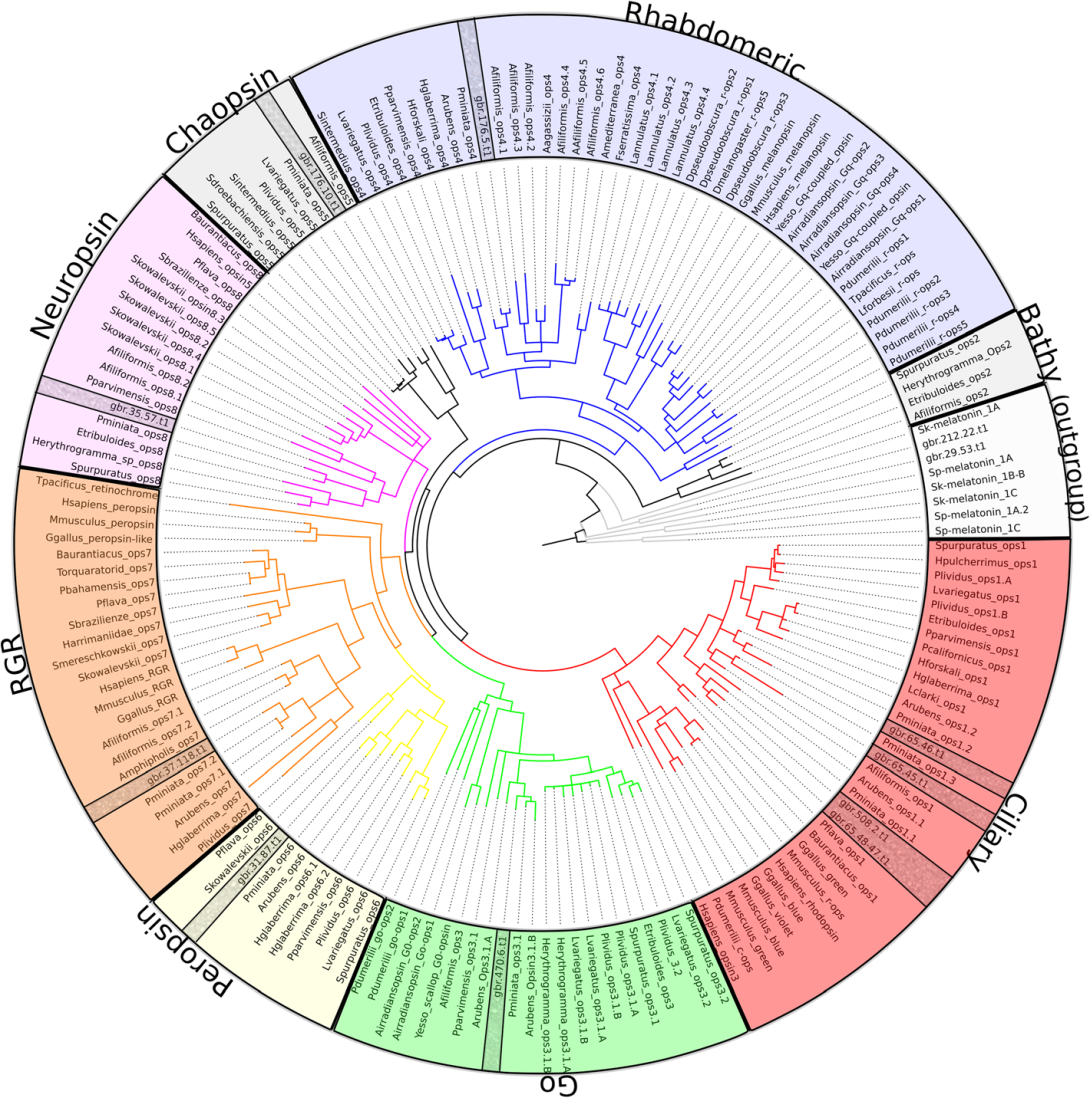
Phylogenomic bayesian tree of 169 opsin sequences with melatonin receptors as the outgroup. There are 10 *A. planci* opsins (bold and checkered background) which classify into 7 different groups: 4 c-opsins, 1 chaopsin, 1 r-opsin, 1 peropsin, 1 RGR opsin, 1 go-opsin, and 1 neuropsin. No bathyopsin was found in *A. planci*, and has yet to be identified in any starfish species. The tree was generated by using Mr. Bayes (v3.2.5) [33] 50 million generations, with the GTR + G amino acid substitution model

We obtained tissue specific transcriptomic data from the locomotive tube feet, the eyes found on the terminal tube feet, the radial nerve, and a mixture of gonad (unsexed), stomach, and epidermis from *A. planci* in biological triplicates in order to determine the differential expression of eye related genes, chiefly opsins. Each library produced 25–32 million reads, one exception (one eye library produced 18 million reads) and mapped the *A. planci* genome at a rate of ~ 63%. Initially we identified all possible opsin genes found in the available *A. planci* Great Barrier Reef genome [30] and classified them. We analyzed *A. planci*’s predicted proteins using a combination of Reciprocal Best Hits (RBH) BLAST against sequences collected from literature and hidden Markov model search with pantherSCORE2.0 [32], which returned thirteen putative opsin genes that share high sequence similarity. Closer examination using the genome revealed that two of these sequences were actually one fragmented opsin (gbr.65.47.t1 and gbr.65.48.t1), reducing the total putative opsins to twelve. We manually edited the sequence, which can be found in the opsin fasta file of Additional file 1. These sequences were included in a phylogenetic analysis totaling 169 sequences spanning 40 different species (see Additional file 1). Our phylogenetic analysis, from both Mr. Bayes [33] (Bayesian) and iqtree [34] (maximum-likelihood), revealed ten *A. planci* opsins belonging to 7 groups and two sequences clustering with the melatonin receptors outgroup (Fig. 2; Additional file 2: Figure S1): 1 rhabdomeric opsin (r-opsin), 4 ciliary opsins (c-opsins), 1 peropsin, 1 Go-opsin, 1 RGR opsin, 1 neuropsin, and 1 chaopsin. Chaopsins were first classified as echinoderm specific [7, 16] but were later found to group with several cnidarian opsins [8]. *A. planci* possesses representatives of all previously identified echinoderm opsin groups with the exception of bathyopsin (former echinopsin A), which thus has yet to be identified in any starfish. In all cases, *A. planci* opsins grouped closest with those of *Patiria miniata*--an eyeless starfish--followed by *Asteria rubens* opsins.

**Fig. 3:**
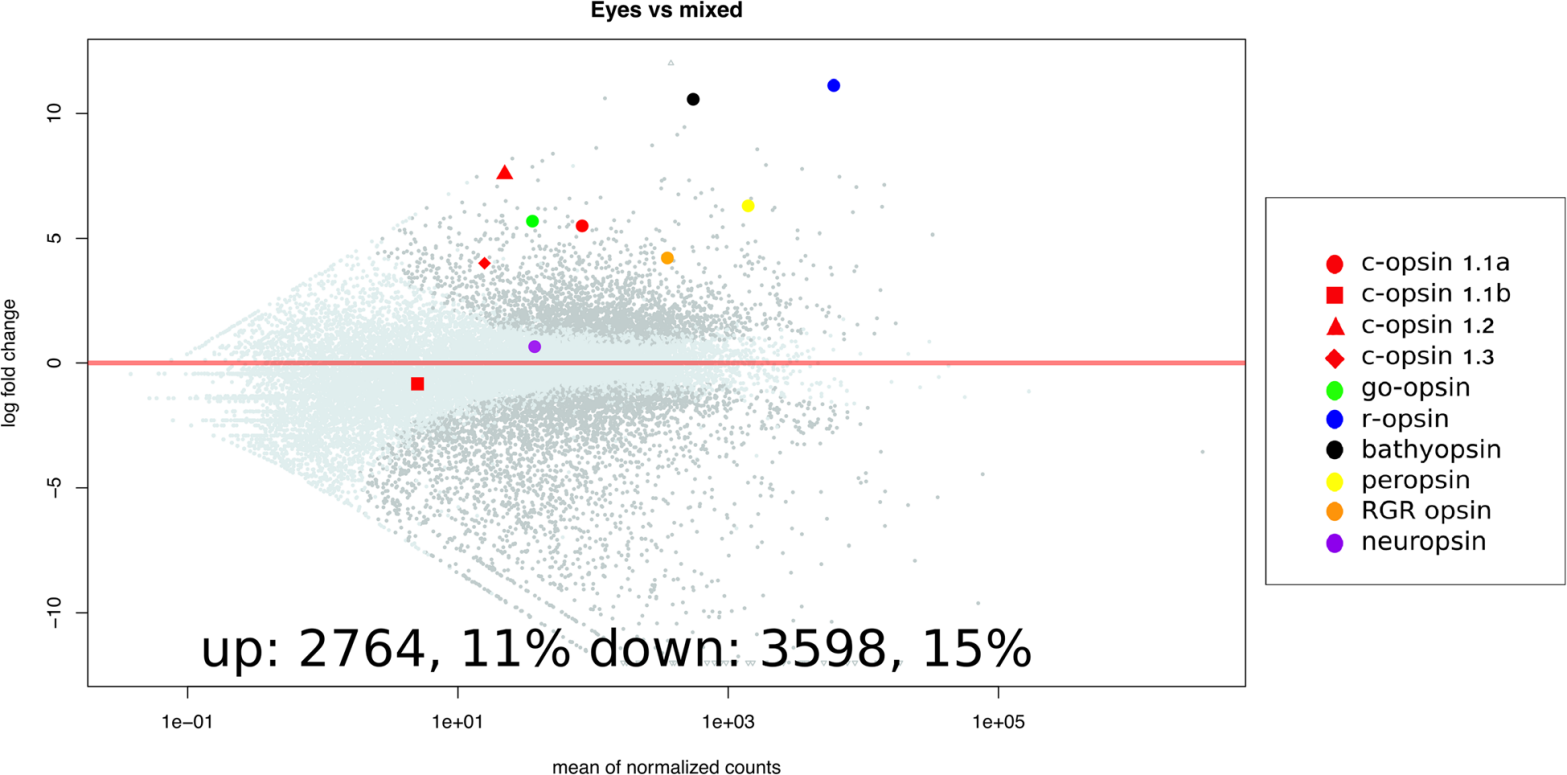
Differential expression of the eye tissue samples versus the mixed tissue samples. The y-axis shows log2 fold change of gene expression, with points greater than 0 being more highly expressed in the eye tissue samples compared to the mixed tissue samples and the x-axis are mean normalized counts of the 3 replicates for each transcript. Points in pale blue have an adjusted *p*-value greater than 0.05, while points in grey are said to be significantly differentially expressed, having an adjusted p-value less than 0.05. Points of different shapes and colors represent the various opsins. R-opsin is the most differentially expressed, followed by chaopsin. With the exception of c-opsin 1.1b and neuropsin, all opsins are significantly differentially expressed in the eyes of *A. planci* compared to the mixed tissue samples

Reads were quasi-mapped to the available *A. planci* transcriptome using Salmon (v0. 82) [35], and the differential expressed genes were identified using DESeq2 [36] with Wald test and were identified as significantly differentially expressed if they had an adjusted *p*-value of 0.05. We compared the eyes, tube feet, and radial nerves to the mixture of gonad, stomach, and epidermis. Each tissue was sequenced in biological triplicates. Of 24,409 transcripts 2414 (9.9%), 2764 (11%) and 2719, (11%) were significantly higher expressed (adjusted *p*-value < 0.05) in the tube feet, eyes and radial nerve compared to the mixture. The number of transcripts that had lower expression than in the mixed tissue, was 2839 (12%), 3598 (15%) and 3490 (14%) in the tube feet, eyes and radial nerve respectively. In *A. planci* eyes, all opsins with the exception of ciliary opsin 1.1b and neuropsin showed higher expression in comparison to the mixed tissues (Fig. 3). This was also the case when comparing opsin expression in the radial nerve to the mixed sample but to a lesser degree (Additional file 3: Figure S2a). Expression of opsins in the tube feet was similar to the mixed tissue for 6 of the opsins but for c-opsin 1.1a, c-opsin 1.1b, c-opsin 1.3 and neuropsin. There was significantly lower expression (adjusted p-value < 0.05) in the tube feet (Additional file 3: Figure S2b). Additionally, we preformed qPCR on several opsins and the neural gene *synaptotagmin 1* (*syt1*) to support out RNA-seq findings (Additional file 4: Figure S3).

In order to assess the putative functionality of the *A. planci* identified opsin sequences, an analysis of the key residues necessary for opsin function was performed (Table 1). In most opsins, the retinal binds to the Lysine K296 via a Schiff base bond, however the proton in the opsin protein is unstable and a counter ion is needed and often supplied by the highly conserved Glutamic acid (E113) residue. There are cases, however, where this residue is replaced by a Tyrosine (Y), Phenylalanine (F), Methionine (M), or Histidine (H) and the other highly conserved Glutamic acid residue, E181, serves as the counter ion [37]. This is the case with the majority of the opsins in *A. planci*, where E113 are replaced with a Tyrosine, with the exception of Ap-Go-opsin which has an Isoleucine (I) in the position 113.

**Table 1:** Analysis of known typically highly conserved key residues and amino acid motifs

*A. planci* Opsin	Disulfide bond^a^ (C110/C187)	LxxxD^b^ (79)	Counterion^c^ (E113/E181)	Schiff base^d^ (K296)	NPxxY(302)
C-opsin 1.1a (gbr.65.47.t1)	C/C	ICVAD	Y/E	K	NPVIY
C-opsin 1.1b (gbr.508.2.t1)	C/C	VCVAD	Y/−	K	NPVIY
C-opsin 1.2 (gbr.65.46.t1)	C/F	ISVGD	Y/−	R	N----
C-opsin 1.3 (gbr.65.45.t1)	L/C	VCEA-	Y/A	K	NPIIY
Go-opsin (gbr.470.6.t1)	C/C	MAVSD	I/E	K	NPLIY
R-opsin (gbr.176.5.t1)	C/C	LAFSD	Y/E	K	NPLVY
Chaopsin (gbr.176.10.t1)	C/C	LSGSD	Y/E	K	NPIIY
Peropsin (gbr.31.87.t1)	C/C	ASAGD	Y/E	K	NPLMF
RGR opsin (gbr.37.113.t1)	C/C	LCAGD	Y/E	K	NAALQ
Neuropsin (gbr.37.57.t1)	C/C	LAVSD	Y/E	K	NPIIY

^a^Motif required for recognition of rhodopsin by G-protein [87]

^b^Motif interacting with NPxxY motif upon receptor activation for structural constraints [88]

^c^Glutamic acid residues stabilizing the Schiff base bond

^d^Lysine residue forming Shiff base bond with retinal Motif providing structural constraints in response to photoisomerization during formation of the G protein-activating Meta II [89]

In addition to ten opsin sequences, we have also observed ten *A. planci* sequences that are potential G protein alpha subunits. Phylogenomic methods classified these sequences as 3 Gα_s_, 1 Gα_o_, 4 Gα_i_, 1 Gα_q_, and 1 Gα_12_ (Additional file 5: Figure S4). All identified G protein alpha subunits with the exception of 1 Gα_s_ (gbr.231.19.t1), 1 Gα_i_ (gbr.143.10.t1) and the Gα_12_ are higher expressed in the eyes of *A. planci* compared to the mixed tissue samples (Additional file 6: Fgure S5).

### Ciliary and rhabdomeric opsins

**Fig. 4:**
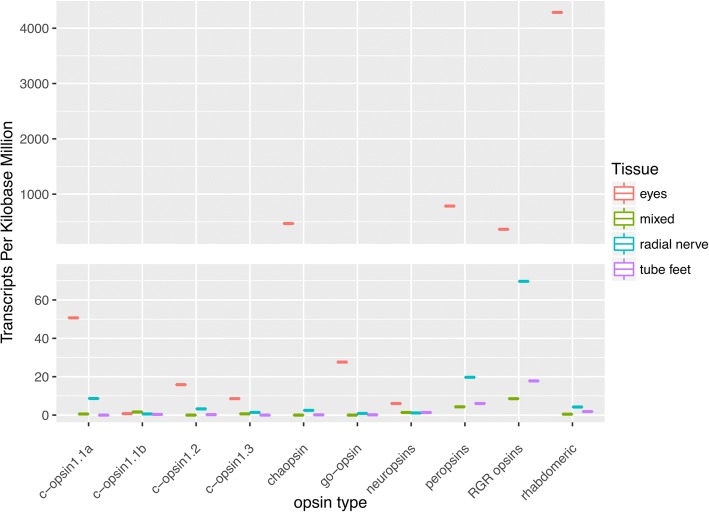
Summary of differential opsin expression data from the four different *A. planci* tissue samples. Counts were normalized for transcripts length and library size using transcripts per kilobase million (TPM) in order to compare opsins for each tissue type. For eyes (red) the highest expressed opsins are r-opsin, peropsin, chaopsin, RGR opsin, and go-opsin, with c-opsin 1.2, c-opsin 1.3, and neuropsin being expressed at low amounts, while no expression is observed in c-opsin 1.1b. RGR opsin and peropsin were the highest expressed amongst the other tissues. The mixed tissue (green) and tube feet (purple) have little to no expression (TPM < 0.5) of c-opsin 1.2, c-opsin 1.3, go-opsin, and chaopsin

There are four ciliary opsins identified in the *A. planci* genome, three of which are expressed in the eyes; c-opsin 1.1a, 1.2 and c-opsin 1.3. These three c-opsins were closely clustered on gbr_scaffold65 (~ 70 kb), and observed to be significantly differentially expressed in the animal, as neither are expressed in the mixed tissue (Fig. 4; Additional file 4: Figure S3). Of the Ap-c-opsins, 1.1a is the highest expressed, followed by Ap-c-opsin 1.2 and 1.3. C-opsin 1.2 was observed to not have the K296 residue, which is required for the formation of the Schiff base, but instead an Arginine (R) residue is found in this position. This is the only *A. planci* opsin missing this key residue. While the E113 counter ion is replaced with Tyrosine (Y) in all of the Ap-c-opsins, the E181 counter ion is present in Ap-c-opsin1.1a, completely missing in Ap-c-opsin 1.1b and 1.2 and replaced with an Alanine (A) in Ap-c-opsin 1.3. The Ap-c-opsin 1.2 and Ap-c-opsin 1.3 are missing other important motifs, the C187 and C110 disulfide bond motifs [38], respectively (Table 1).

*A. planci*’s r-opsin, on the other hand, is the most highly differentially expressed of the opsins and of any other genes when comparing eyes with the mixed tissue (Fig. 3). Further, the Ap-r-opsin was observed to be the most highly expressed in the starfish eye and its sequence features the Lysine residue (K296), critical for the Schiff base formation, and a putative counter ion (E181) (Table 1).

### Chaopsin

Chaopsin is a recently identified group of opsins. Ramirez et al. [8] found the formerly described groups of anthozoa I opsins [39] and the echinoderm echinopsin B [7] to cluster forming the chaopsin group. In agreement with D’Aniello et al. (2015) we found an *A. planci*’s chaopsin to cluster together with other ambulacrarian chaopsins between the r-opsin and c-opsin clades (Fig. 2). Ap-chaopsin is amongst the highest differentially expressed opsins in our *A. planci* transcriptomes, with ~ 9.7 log_2_ fold changes in the eye compared to the mixed tissue (Fig. 3). It is also expressed in the radial nerve, but to a far lesser degree, and are not significantly expressed in the mixed tissues or the tube feet (Fig. 4 and Additional file 3: Figure S2).

### Peropsin RGR and go opsins

Peropsin and RGR opsin are the highest expressed of the opsins in the tube feet, mixed, and radial nerve, although both still have higher expression in the eyes (Fig. 4 and Additional file 3: Figure S2). The disulfide bond linkage C110/C187, counterion sites and the Lysine for the Schiff base formation are all present. However, both *A. planci* RGR-opsin and peropsin contain variations of the NPxxY motif, NAALQ and NPLMF, respectively (Table 1). Additionally, peropsin has a variation of the LxxxD motif, ASAGD. Ap-Go-opsin was expressed in both the eyes and radial nerve but was not found in the mixed sample or in the tube feet (Figs. 3 and 4).

## Discussion

Light sensing is an important aspect of life and much of it is mediated or initiated by the G-protein-coupled receptor proteins, opsins. The release of the annotated draft genome of *A. planci* has prompted us to investigate its opsin repertoire and expression in a tissue specific manner. This allowed us to classify the specific opsins and to infer possible function and further expand the knowledge of opsin evolution especially within deuterostomes. Ten opsins were identified spanning seven clades: r-opsin, c-opsin, Go-opsin, peropsin, neuropsin, RGR opsin and chaopsin. Opsins have also been sequenced from two other starfish species, *Asterias rubens* and *Patiria miniata*. Through a phylogenomic analysis, it was observed that *A. planci* opsins grouped closest to those in the eyeless *P. miniata*. This grouping is in accordance with the phylogenetic position of these starfish species [40], with *A. planci* as an Acanthasteridae more closely related to *P. miniata*, an Asterinidae, (both species belonging to Valvatida)- than to *A. rubens* belonging to Forcipulatacea. However, studies on tissue specific opsin expression are needed to reveal in which organs of the eyeless starfish *P. miniata* the respective opsin orthologs are expressed. To this point, it remains unclear if opsins potentially serving a visual function in the eye possessing *A. planci* might have switched functions in the eyeless representative. Alternatively, opsin expression in *P. miniata* might simply persist as a potential evolutionary remain, a finding known e.g. from blind cave salamanders. Their photoreceptors remain to express opsins despite the apparent loss of shading pigmentation and their highly degenerated morphology [41].

### Rhabdomeric opsin

Electrophysiological recordings from the photoreceptors of *A. planci* have strongly suggested light absorption here to be utilizing a single opsin. Of the several opsins found to be expressed in the eyes of *A. planci*, r-opsin was the highest and most differentially expressed gene in the eye transcriptome when compared to mixed samples, thus suggesting this to be the opsin utilized for vision. Whereas rhabdomeric opsins have been described in many protostome species as the primary opsin for vision (reviewed in [42]) and as a non-visual opsin (melanopsin) in vertebrates (reviewed in [31]), no deuterostome eye has previously been found to express an r-opsin for primary vision. In the sea urchin *S. purpuratus* r-opsin in tube feet has been proposed to be involved in a visual context facilitating phototaxis but not in spatial vision [14], *A. planci* eyes have been previously demonstrated to perform proper spatial vision [25–27]. Our findings thus provide first indications for a deuterostome eye utilizing an r-opsin for spatial vision and might facilitate in identifying the split between r-opsin and c-opsin in complex eyes. However, those findings await final confirmation by future expression studies.

### Ciliary opsin

**Fig. 5:**
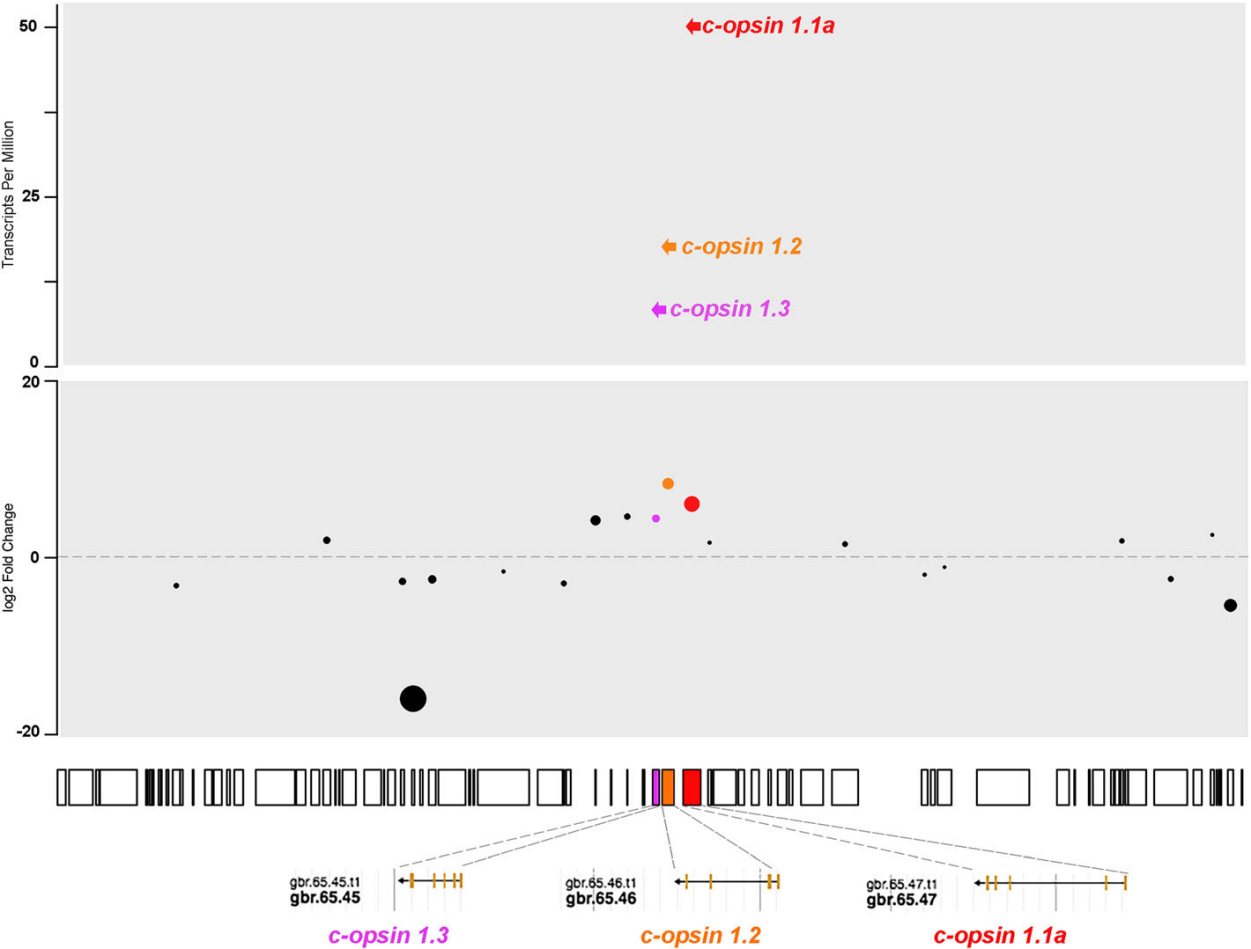
Three of four c-opsins found on same scaffold in decreasing order of expression. The boxes below the two panels represent “gbr_scaffold65” from the Great Barrier Reef assembly of the *A. planci* genome. Each box represents a gene and the size of the box correlates to the size of the gene. Three of the four Ap-c-opsins are located on the same scaffold all being transcripted 3′ to 5′; Ap-c-opsin 1.1a (red), Ap-c-opsin 1.2 (orange) and Ap-c-opsin 1.3 (purple). Bottom panel: The log2 fold change of significantly differentially expressed genes (FDR < 0.05) comparing *A. planci* eyes the mixed tissue samples, with the three Ap-c-opsins highlighted. Top panel: Expression of the three Ap-c-opsins clustered on “gbr_scaffold65”, the expression level correlates with the spatial ordering within the cluster (in the direction 3′ to 5′), the most 3′ having the highest expression and the most 5′ having the lowest expression

Ciliary opsins are well known for their role in vertebrate vision and brain function in some invertebrates [43]. They are differentially expressed in *A. planci* eyes, with the exception of Ap-c-opsin 1.1b, which is not expressed in the eye and does not cluster on the same scaffold as the other 3 Ap-c-opsins. Interestingly, the three clustered opsins show a correspondence between their spatial ordering within the cluster (in the direction 3′ to 5′) and their expression level, with most 3′ Ap-c-opsin being the most highly expressed in both eye and radial nerve (Fig. 5). C-opsins appear clustered also in vertebrates, for example, human’s medium and long wavelength ciliary opsins (opsin1MW1, opsin1MW2, opsin1MW3 and opsin1LW) are clustered on chromosome X, while human’s short wavelength c-opsin is on chromosome 7 (Ensembl genome browser; [44]). Clustering of opsins has so far not been described outside deuterostomes, but it is also seen in the echinoderm, *S. purpuratus* where Go-opsins (opsin 3.1 and 3.2) clustered on scaffold26 (Echinobase; [45]). The significance of the occurrence of opsin clustering in echinoderms is not obvious and awaits more functional studies.

### Chaopsin

Chaopsin (Ap-chaopsin) is the second most differentially expressed opsin in *A. planci* eyes and it has many of the motifs necessary for phototransduction, including the NPxxY binding motif in the 7th transmembrane domain involved in coupling with the G protein. Little is known about the functions of chaopsins (or opsin5) but they have been identified in Echinoidea, Asteroidea, and Ophiuroidea [7, 19, 45–47]. Chaopsins have been found analyzing genomic data or transcriptomes of hypothesized photosensitive tissues, such as the tube feet in *Strongylocentrotus droebachiensis* [46] and *Strongylocentrotus intermedius* [47]. A chaopsin was identified in the genome of the eyeless *P. miniata* but was not found in transcriptome data of the eye possessing starfish *A. rubens*. The lack of chaopsin in *A. rubens* is probably a methodological artifact, since general arm tissue, including radial nerve and tube feet, but not eyes, has been used to generate these transcriptomic data [48]. In the Caribbean elkhorn coral, *Acropora palmata,* opsin3, which together with echinoderm opsin5 belongs to the chaopsin clade [8], has been demonstrated to couple with a Gq-protein in a light-dependent manner [5]. This leads us to hypothesize that the Ap-chaopsin may be important for phototransduction in *A. planci* and potentially in all echinoderms. The exact role of this opsin remains elusive, though.

### Peropsin and RGR opsin

Peropsin and RGR opsin were expressed in the tube feet, the mixed tissue, and the radial nerve of *A. planci*, but both have the highest expression in the eyes. They are considered as photoisomerase enzymes and not as photopigments, since they bind to all-trans retinaldehyde to regenerate 11-cis-retinoids for pigment regeneration. This has been observed for peropsin in the vertebrate retinal pigment epithelium [49], in cephalopod photoreceptors [50] and in the jumping spider [51]. Knock-down mice [52], together with biochemical and spectroscopic studies in amphioxus [53], have demonstrated the same properties for RGR opsin in chordates. This family of opsins is thus important for visual pigment regeneration [54]. While RGR opsins are known to not have the NPxxY binding motif in the 7th transmembrane domain involved in coupling with the G protein, this motif is often found in peropsins [51, 53]. This was not the case in *A. planci*, where both RGR opsin and peropsin have varying NPxxY binding motifs. This could alter peropsin’s ability to couple with G proteins, further supporting its function as photoisomerase. However, it is worth mentioning that, in chicken, the presence of both peropsin and RGR opsin is thought to serve in the visual cycle of the circadian clock [55].

### Go-opsin

In *A. planci* eye and radial nerve transcriptomes, we found expression of a Go-opsin along with putative Go alpha subunit proteins. Evidence for Go-opsins in animals is rare. These opsins interact with a specific G-protein that differs from those in the c-opsin as well as the r-opsin transduction cascade [6, 56]. In the retina of the bivalve *Patinopecten yessonensis*, Go-opsin is expressed in a layer of ciliary photoreceptor cells, which do not express a ciliary opsin [57]. However, in the marine annelid *Platynereis dumerilii*, Go-opsin is co-expressed with two r-opsins in the photoreceptor cells of the larval eye [58]. Knocking down Go-opsin did not lead to absence of phototaxis in *P. dumerilii* but did reduce the sensitivity to the blue-cyan part of the color spectrum (λ_max_ = 488 nm). A similar absorbance spectrum (λ_max_ = 483 nm) was observed in amphioxus, *Branchinostoma belcheri*, Go-opsin [53, 59]. While the morphological and expression data on Go-opsin in *P. yessonensis* point towards a functioning in a visual context, no other studies have found this. Our data does not reveal if *A. planci* Go-opsin is co-expressed with any other opsins inside the same cells, nonetheless, the fact that this opsin is expressed in the starfish eye along with the results from annelids and amphioxus, opens up the possibility that it is involved in spectral tuning of vision in *A. planci*. Such a tuning would indeed be consisted with the spectral sensitivity curves obtained by electrophysiology in this starfish species [26].

## Conclusion

In conclusion, our findings demonstrate that the eye of the starfish *A. planci* expresses an entire set of ten different opsin proteins. This starfish has recently been demonstrated to possess spatial vision utilized when locating its preferred coral food. Ap-r-opsin is by far the most highly expressed photopigment in its eyes, and thus likely to be the photopigment facilitating these sophisticated visually guided behaviours. *A. planci* is thus one more echinoderm possessing a much more complex photoreceptor system than previously assumed and the variety of opsins found differentially expressed in various starfish tissues by our transcriptomic analyses sets the ground for comparative studies on evolutionary changes in photoreceptor function that occurred towards the vertebrate eye. In addition to investigating vision in *A. planci* this study thus provides tissue specific transcriptome data that will aid in future evolutionary studies.

## Material and methods

### Animals

The specimens of *A. planci* used in this study were hand collected on the Great Barrier Reef off the coast of Cairns, Australia. After collection the animals were kept in holding tanks with running seawater at 26 degrees for 2–3 days and then flown to Denmark. In Denmark they were kept under similar conditions at the Danish National Aquarium, The Blue Planet, where they were fed three times a week with a past of enriched squid and fish meat. Tissue samples were taken from four specimens with diameters of 15–23 cm. Three eyes which were dissected from the terminal tube feet, 3 eye-less locomotory tube feet, approx. 5 cm radial nerve, and pieces of the gonads, the stomachs, and the epidermis were sampled from each of the four specimens and stored in RNAlater at 4 °C. Even though microdissection was performed the eyes will likely contain some remains of the modified tube foot it is situated on. The samples of the radial nerve will also contain the epithelium covering the nerve ventrally. Two additional animals were collected at the coastal reefs of Guam and a total of 12 eyes and 10 cm radial nerve were taken from them directly after collection and stored in RNAlater at 4 °C.

### RNA extraction and sequencing

The tissue samples were removed from the RNAlater, immediately frozen with liquid nitrogen and homogenized using a mortar and pestle. Powdered tissues were then dissolved in EuroGOLD RNAPure (EMR 506100) and processed using EUROzol RNA extraction protocol (EMR055100, euroclone), then subjected to LitCl (4 M) purification. Library preps and sequencing were done at Università degli Studi di Salerno in biological triplicates using SMART-Seq v4 Ultra Low Input RNA Kit to ensure enough RNA was available for each library. 10 ng of RNA was used for each library and we used 8 PCR cycles to amplify the samples.

Sequenced reads were examined using fastqc and then quality filtered and trimmed using trimmomatic (v0.33) [60]. Quality controlled reads were quasi-mapped and quantified to v1 Great Barrier Reef *Acanthaster planci* transcriptome using salmon (v0. 82) [35]. Transcripts per million (TPM), the normalized transcript counts [61]. Differentially expressed genes were identified using DESeq2 [36]. Wald test and transcripts were identified as significantly differentially expressed if they had an adjusted *p*-value ≤0.05, and − 1.5 ≥ log2FC ≥ 1.5. All scripts can be found at https://github.com/elijahlowe/Acanthaster_opsins.git. Additionally, reads were mapped onto the *A. planci* genome to determine mapping rates using transrate [62] default setting.

### Identification of opsin sequences

Opsin protein sequences were collected from echinoderms [7, 19, 46, 47, 63–66], hemichordates [67], molluscs [56, 68, 69], arthropods [70, 71], vertebrates [55, 72–77] and annelids [78] covering 40 species and 159 opsin sequences. Additional 7 melatonin receptor sequences were used as outgroup. These sequences were retrieved from various databases including Echinobase [45], and NCBI [79], as well as from publications themselves, as described in Additional file 1. These sequences were used to perform Reciprocal Best Hits (RBH) BLAST against the *A. planci* (GBR) predicted proteins. Additionally, pantherSCORE2.0 [32] was used against the *A. planci* predicted proteins to identify opsin sequences using hidden Markov models. The collected sequences were aligned with MAFFT (v7.215) [80, 81] using L-INS-i algorithm which is better designed for divergence sequences and performed well when benchmarked against other multiple sequence aligners [82]. The aligned sequences were then trimmed using trimAl [83], removing gaps that occurred in 10% of the alignments while being sure to retain 60% of the total sequence length. Maximum likelihood phylogenetic trees were generated using the aligned sequences with iqtree [84] with 10,000 ultrafast bootstrap support [34] using the ‘a Bayesian-like transformation of aLRT’ (abayes) method [85], and GTR + G amino acid substitution model and visualised with figtree (v1.4.3) [86]. Additional trees were then generated using bayesian tree was then generated using selected using MrBayes (v3.2.5) [33] 50 million generations, with the GTR + G amino acid substitution model as well. Modifications such as additional labels and visual effects were done using inkscape.

### Reverse transcription

First-strand cDNA was synthesized in a 20 μl reaction from 100 ng of total RNA using the SuperScript VILO cDNA synthesis kit (Invitrogen). The synthesis of the cDNA was performed with the following program: 25 °C for 10 min, 42 °C for 90 min, 85°C for 5 min. Specific primer sets for each gene were designed to amplify products 100–200 bp in length using the Primer3 program (Rozen and Skaletsky, 2000). Primers’ sequences were as follows: syt1forward, cgacccctacgtcaaagtgt; syt1 reverse, gacctcaccaatctggtcgt; Ef1a forward, ggtcattggccacgtagact; Ef1a reverse, gctctgccttcaacttgtcc; c-opsin forward, gctatcctggcgctgtactc; c-opsin reverse, tcagtgtgctcggtaggatg; r-opsin forward, cagatcgccaaagtgggtat; r-opsin reverse, gcggaactcttggctaacac; chaopsin forward, cttcctgtcagcctggactc; chaopsin reverse, aactgtgggctcatcaatcc.

### Real time QPCR

Real time QPCR amplification was performed with diluted cDNA (1:4 dilution). Each 10 μl reaction contained 5 μl of SYBR Green reagent, 4 μl of forward and reverse primer mix (0,75 μM each) and 1 μl of 1:4 cDNA. Reactions were performed in technical triplicate with three different biological samples using the ViiA 7 REAL TIME PCR with SYBR Green chemistry (Applied Biosystems). The cycling condition was: 95 °C for 20 s, 40 cycles at 95 °C for 1 s and 60 °C for 20 s, 95 °C for 15 s, 60 °C 1 min, and a gradient from 60 °C to 95 °C for 15 min. All primer pairs were validated by QPCR against a positive (genomic DNA) and negative (water) control.

## Additional files

Additional file 1: Fasta file containing all 169 opsin and melatonin sequences used to generate phylogenetic tree. (FA 60 kb)

Additional file 2: **Figure S1.** Bayesian tree of opsins sequences with support. Tree was generated using using MrBayes (v3.2.5) [33] 50 million generations, with the GTR + G amino acid substitution model. (PNG 4279 kb)

Additional file 3: **Figure S2.** Differential gene expression in all *A. planci* tissue samples with the opsins highlighted: c-opsins in red, go-opsins in green, chaopsin in black, neuropsin in purple, peropsin in yellow, r-opsin in blue and RGR opsin in orange. The y-axis in the log_2_ fold-change, as the distance from the y-axis increases the more differentially expressed a gene is in one tissue versus the other. The x-axis represents counts per million (CPM), an increase on this axis shows genes with more reads counts. (PNG 1039 kb)

Additional file 4: **Figure S3.** Graphical representation of QPCR results. a) Relative expression to the elongation factor *Ef1A* gene of *synaptotagmin 1* (*syt1*), *r-opsin*, *chaopsin* and *c-opsin1a* genes in mixed tissues, tube feet (TF), terminal tube feet (eye) and radial nerve (RN). Calculations from QPCR raw data used the formula 1.9^-ΔCt^, where 1.9 is the multiplier for amplification per PCR cycle, and ΔCt is the threshold cycle difference with *Ef1a* found for that sample. b) Fold enrichment of *syt1*, *c-opsin1a*, *r-opsin* and *chaopsin* genes in tube feet (TF), terminal tube feet (eye) and radial nerve (RN) compared to the mixed tissues. Calculations from QPCR raw data used the formula 1.9^-ΔΔCt^, where 1.9 is the multiplier for amplification per PCR cycle, and ΔΔCt is the ΔCt difference between mixed tissues and the other tissues. Data for each gene were normalized against the housekeeping *Ef1a*. All quantitative measurements were done in triplicate on the cDNA obtained from each biological replica tissue sample. Average calculations over the three technical replicas ± standard deviations are reported for each gene with error bars. (JPG 244 kb)

Additional file 5: **Figure S4.** Phylogenomic tree of 92 G-protein alpha subunit sequences. *A. planci* sequences ID’s are highlighted in red. Of the 10 *A. planci* sequences 3 classified as Gα_s_, 1 as Gα_o_, 4 as Gα_i_, 1 as Gα_q_, and 1 as Gα_12_. (JPG 308 kb)

Additional file 6: **Figure S5.** Differential gene expression in the *A. planci* eye samples compared to mixed tissues, with the G protein alpha subunits highlighted: 3 Gα_s_ (red), 1 Gα_o_ (green), 4 Gα_i_ (black), 1 Gα_q_ (purple), and 1 Gα_12_ (yellow). All identified g protein alpha subunits with the exception of 1 Gα_s_ (gbr.231.19.t1), 1 Gα_i_ (gbr.143.10.t1) and the Gα_12_ are show higher expression in the eyes of *A. planci* compared to the mixed tissue samples. (JPG 462 kb)

## Abbreviations

A rubens: *Asterias rubens*
Ap: *Achanthaster planci*
c-opsin: Ciliary opsin
COTS: Crown-of-thorns starfish
CPM: Counts per million
F: Phenylalanine
GBR: Great Barrier Reef
H: Histidine
I: Isoleucine
K: Lysine
M: Methionine
*P. miniata*: *Patiria miniata*
R: Arginine
RBH: Reciprocal Best Hits
r-opsin: Rhabdomeric opsin
TPM: Transcripts per million
Y: Tyrosine

## Footnotes

1 COTS is thought to be divided into four separate species distinguished by bodies of water [28]--Red Sea, the Pacific, the Northern and the Southern Indian Ocean, reclassifying the Pacific ocean species as *Acanthaster solaris* [29]. However, for consistency with the released genome we will refer to the GBR species as *A. planci*.
