# Supplementary figures and images for: The crowns have eyes: multiple opsins found in the eyes of the crown-of-thorns starfish Acanthaster planci
Source: BMC Evol Biol. 2018 Nov 12;18:168. doi: 10.1186/s12862-018-1276-0 (PMC6233551; doi:10.1186/s12862-018-1276-0)

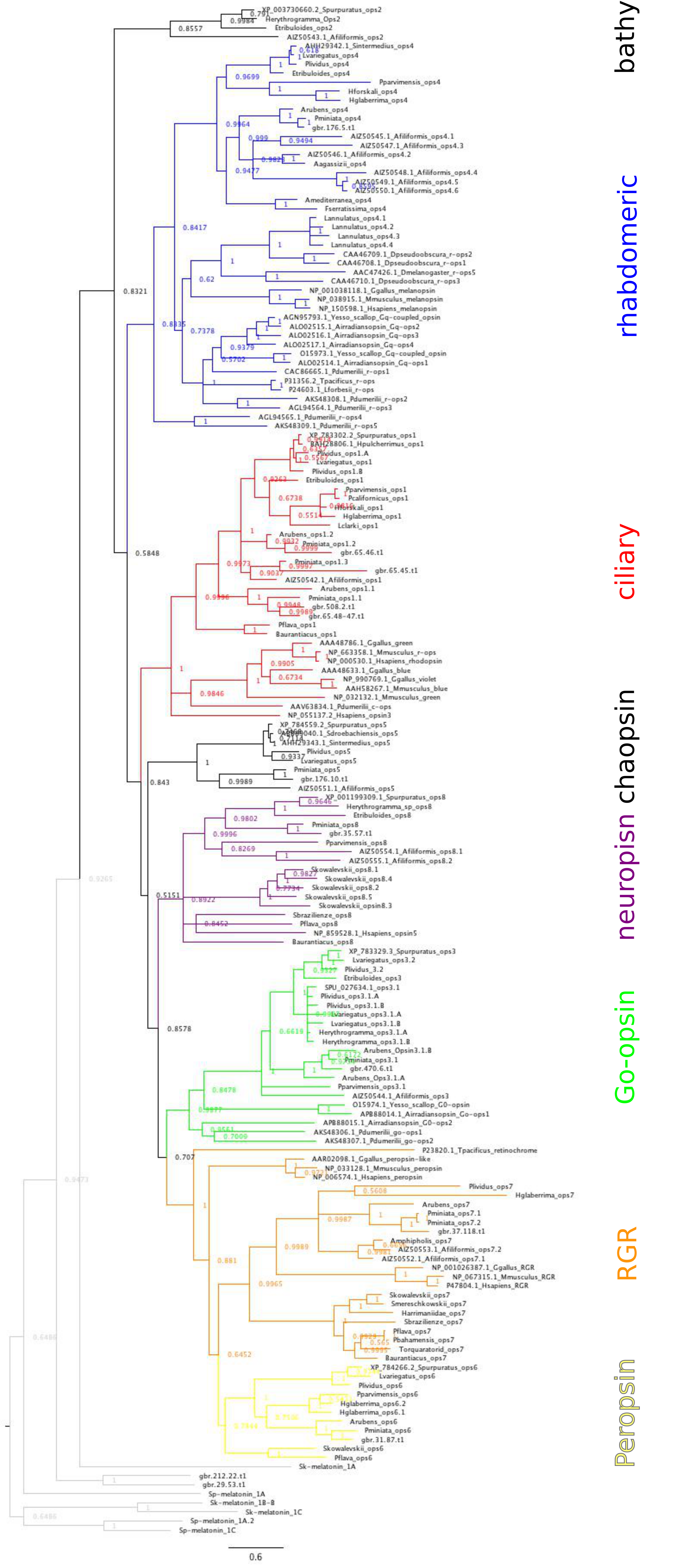

Supplement: Supplementary file 2 — Figure S1. Bayesian tree of opsins sequences with support. Tree was generated using using MrBayes (v3.2.5) [33] 50 million generations, with the GTR + G amino acid substitution model. (PNG 4279 kb) [file 12862_2018_1276_MOESM2_ESM.png]

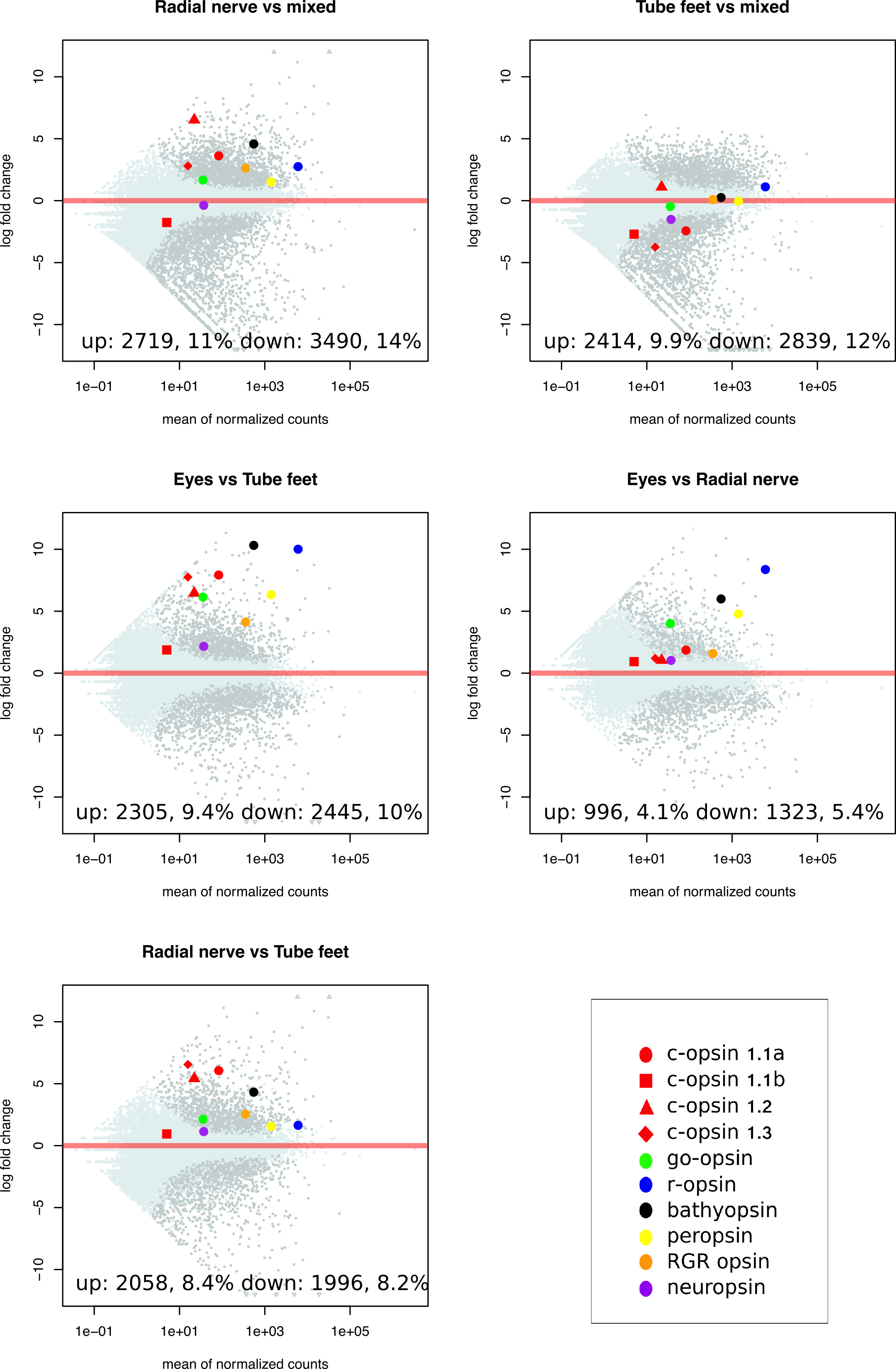

Supplement: Supplementary file 3 — Figure S2. Differential gene expression in all A. planci tissue samples with the opsins highlighted: c-opsins in red, go-opsins in green, chaopsin in black, neuropsin in purple, peropsin in yellow, r-opsin in blue and RGR opsin in orange. The y-axis in the log2 fold-change, as the distance from the y-axis increases the more differentially expressed a gene is in one tissue versus the other. The x-axis represents counts per million (CPM), an increase on this axis shows genes with more reads counts. (PNG 1039 kb) [file 12862_2018_1276_MOESM3_ESM.png]

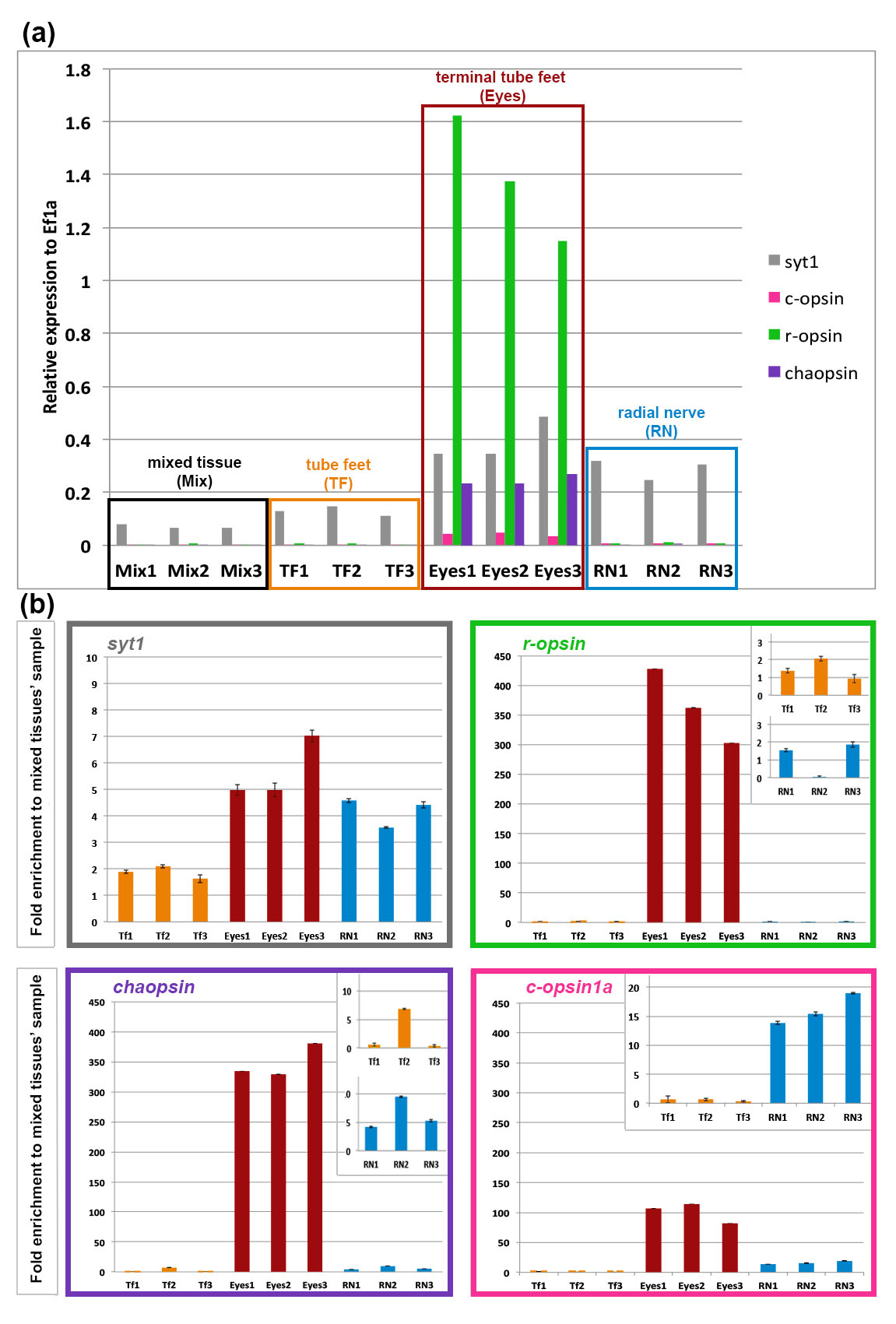

Supplement: Supplementary file 4 — Figure S3. Graphical representation of QPCR results. a) Relative expression to the elongation factor Ef1A gene of synaptotagmin 1 (syt1), r-opsin, chaopsin and c-opsin1a genes in mixed tissues, tube feet (TF), terminal tube feet (eye) and radial nerve (RN). Calculations from QPCR raw data used the formula 1.9-ΔCt, where 1.9 is the multiplier for amplification per PCR cycle, and ΔCt is the threshold cycle difference with Ef1a found for that sample. b) Fold enrichment of syt1, c-opsin1a, r-opsin and chaopsin genes in tube feet (TF), terminal tube feet (eye) and radial nerve (RN) compared to the mixed tissues. Calculations from QPCR raw data used the formula 1.9-ΔΔCt, where 1.9 is the multiplier for amplification per PCR cycle, and ΔΔCt is the ΔCt difference between mixed tissues and the other tissues. Data for each gene were normalized against the housekeeping Ef1a. All quantitative measurements were done in triplicate on the cDNA obtained from each biological replica tissue sample. Average calculations over the three technical replicas ± standard deviations are reported for each gene with error bars. (JPG 244 kb) [file 12862_2018_1276_MOESM4_ESM.jpg]

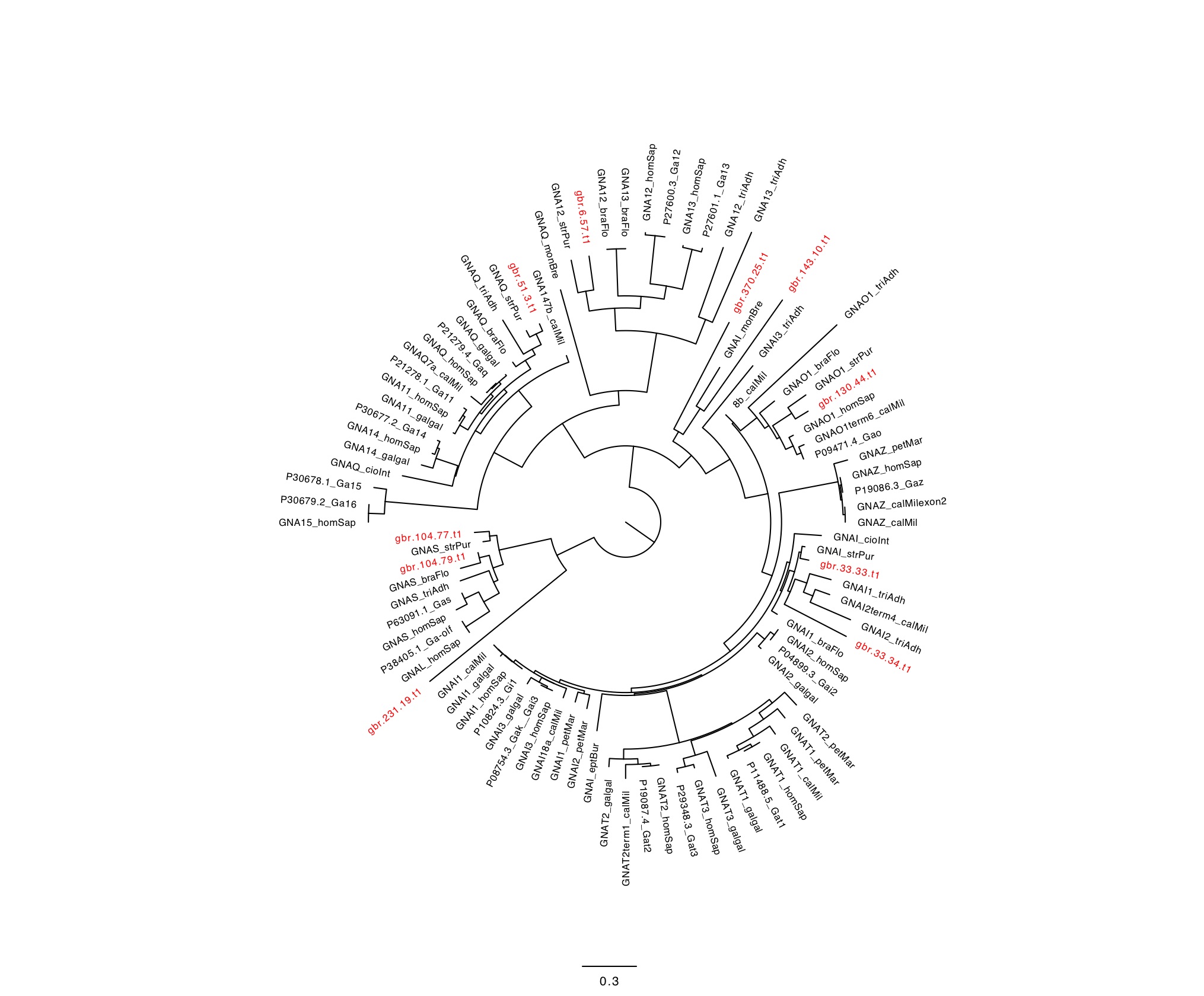

Supplement: Supplementary file 5 — Figure S4. Phylogenomic tree of 92 G-protein alpha subunit sequences. A. planci sequences ID’s are highlighted in red. Of the 10 A. planci sequences 3 classified as Gαs, 1 as Gαo, 4 as Gαi, 1 as Gαq, and 1 as Gα12. (JPG 308 kb) [file 12862_2018_1276_MOESM5_ESM.jpg]

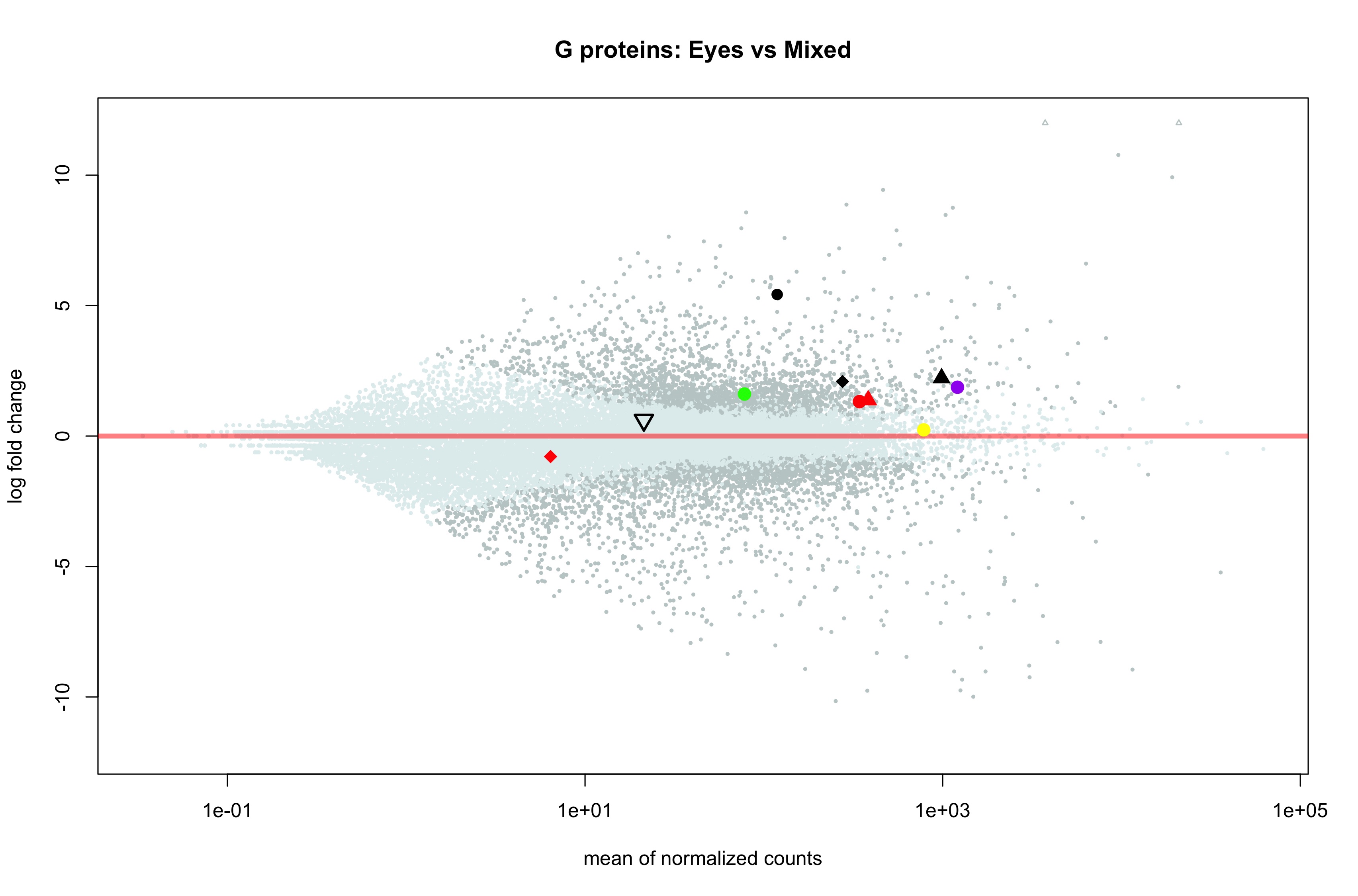

Supplement: Supplementary file 6 — Figure S5. Differential gene expression in the A. planci eye samples compared to mixed tissues, with the G protein alpha subunits highlighted: 3 Gαs (red), 1 Gαo (green), 4 Gαi (black), 1 Gαq (purple), and 1 Gα12 (yellow). All identified g protein alpha subunits with the exception of 1 Gαs (gbr.231.19.t1), 1 Gαi (gbr.143.10.t1) and the Gα12 are show higher expression in the eyes of A. planci compared to the mixed tissue samples. (JPG 462 kb) [file 12862_2018_1276_MOESM6_ESM.jpg]
